# The impact of COVID‐19 upon the delivery of exercise services within cystic fibrosis clinics in the United Kingdom

**DOI:** 10.1111/crj.13484

**Published:** 2022-03-01

**Authors:** Owen W. Tomlinson, Zoe L. Saynor, Daniel Stevens, Don S. Urquhart, Craig A. Williams

**Affiliations:** ^1^ Children's Health and Exercise Research Centre, Sport and Health Science University of Exeter, St Luke's Campus Exeter UK; ^2^ Royal Devon and Exeter NHS Foundation Trust Hospital Exeter UK; ^3^ Physical Activity, Health and Rehabilitation Thematic Research Group, School of Sport, Health & Exercise Science, Faculty of Science & Health University of Portsmouth Portsmouth UK; ^4^ School of Health and Human Performance, Division of Kinesiology Dalhousie University Halifax Nova Scotia Canada; ^5^ Department of Pediatrics, Division of Respirology, Faculty of Medicine Dalhousie University Halifax Nova Scatia Canada; ^6^ Department of Paediatric Respiratory and Sleep Medicine Royal Hospital for Children and Young People Edinburgh UK; ^7^ Department of Child Life and Health University of Edinburgh Edinburgh UK

**Keywords:** coronavirus pandemic, physiotherapy, survey, telehealth

## Abstract

**Objectives:**

The COVID‐19 pandemic has resulted in unprecedent changes to clinical practice, and as the impact upon delivery of exercise services for people with cystic fibrosis (CF) in the United Kingdom was unknown, this was characterised via a national survey.

**Methods:**

An electronic survey was distributed to healthcare professionals involved in the exercise management of CF via established professional networks.

**Results:**

In total, 31 CF centres participated. Findings included significant reductions in exercise testing and widespread adaptation to deliver exercise training using telehealth methods. Promisingly, 71% stated that they would continue using virtual methods of engaging patients in future practice.

**Conclusion:**

These findings highlight adaptation to the COVID‐19 pandemic and the need to develop sustainable and standardised telehealth services to manage patients moving forwards.

**Abbreviations**
CF: cystic fibrosis
MDT: multidisciplinary team
NHS: National Health Service
PA: physical activity
UK: United Kingdom

## 1 INTRODUCTION

Participation in regular physical activity (PA) and exercise is beneficial for people with cystic fibrosis (pwCF) and is an integral part of their clinical management. Regular exercise testing and reviews of exercise training programmes are therefore recommended to occur at least annually.^1^, ^2^

Upon the emergence of the SARS‐CoV‐2 coronavirus‐2019 (COVID‐19) pandemic, restrictions were imposed by the U.K. Government to limit transmission and included additional constraints for clinically vulnerable individuals, such as pwCF.^3^ Specifically, pwCF were asked to ‘shield’ at home, with recommendations to avoid face‐to‐face hospital appointments where possible.^4^ Early evidence from Switzerland reported that such measures negatively impacted upon PA levels of pwCF, with noted barriers including closure of facilities, lack of motivation and cancelled training supervision.^5^

As with many services within the National Health Service (NHS), CF multidisciplinary care teams (MDTs) have been forced to adapt and deliver services virtually where possible (e.g., telephone, video and email consultations).^6^ However, it is unknown how feasible such changes are for CF MDTs and to what extent exercise services in particular have been affected. Given the importance of exercise testing and training for pwCF, identifying how exercise services have changed during the COVID‐19 pandemic is a priority; to ensure high‐quality services are still delivered and identify areas where additional resources are required.

Understanding service change has applicability beyond the current pandemic. Given that pwCF are regularly advised to segregate from one another for infection control reasons, and ‘shield’ at home when unwell, any positive or sustainable changes to clinical practice may be appropriate for continued implementation. This online survey therefore sought to identify the impact of the COVID‐19 pandemic upon the delivery of exercise services within CF clinics in the United Kingdom.

## 2 METHODS

Questions asked within this survey formed part of a wider survey related to exercise services in CF MDTs across the United Kingdom (e.g., frequency of testing and training, equipment and staffing provision, barriers and facilitators to implementation), a replication of a previous survey.^7^ Information concerning centre location, patient population and job role of respondents was collated, alongside specific COVID‐19‐related questions (Table 1). These questions were collaboratively developed by the authorship team, which consists of both clinicians and researchers, and the questionnaire was internally tested by academic colleagues for readability and ease of use prior to dissemination to respondents.

**TABLE 1:** Survey questions related to COVID‐19 and exercise services

Q1	Has the COVID‐19 pandemic affected your ability to deliver exercise testing?				
	YES		NO		
Q2	How often are you able to undertake exercise testing due to the pandemic?				
	ALWAYS	MOST OF THE TIME	ABOUT HALF THE TIME	SOMETIMES	NEVER
Q3	How has you centre adapted exercise testing in light of the pandemic (e.g., video tests, home visits, stopped altogether, no change)^a^				
Q4	Has the COVID‐19 pandemic affected your ability to deliver exercise training?				
	YES		NO		
Q5	How often are you able to undertake exercise training due to the pandemic?				
	ALWAYS	MOST OF THE TIME	ABOUT HALF THE TIME	SOMETIMES	NEVER
Q6	How has you centre adapted exercise training in light of the pandemic (e.g., video tests, home visits, stopped altogether, no change)^a^				
Q7	What have been the major barriers to delivering exercise services (testing and training) during the pandemic?^a^				
Q8	What resources have you found to benefit your team in during the pandemic?^a^				
Q9	Are there any changes you have made due to the pandemic that you intend to keep and/or maintain?^a^				
Q10	What questions have your patients been asking you in relation to exercise and COVID‐19?^a^				
Q11	Have you been able to confidently answer your patients' questions?				
	YES	NO	NO PATIENTS HAVE ASKED QUESTIONS		
Q12	Do you have any questions with regards to exercise and COVID‐19 for cystic fibrosis that you would like answering/addressing?^a^				
Q13	Do you have any final comments on exercise and COVID‐19 in your centre?^a^				

^a^ Indicates questions were free‐text responses.

The survey was distributed via email, by the Association of Chartered Physiotherapists in CF, the U.K. CF and Exercise Technicians Network, and U.K. CF Medical Association to their respective memberships. It was asked that a single member of each MDT (ideally the person responsible for exercise services) completed the survey on behalf of their site, to ensure a single response per centre. The survey was distributed in January 2021 and remained open for 6 weeks, to maximise the response rate. This survey was hosted using an online platform (Qualtrics XM; Provo, Utah, USA), chosen because of its compatibility with both computers and smartphones, whilst also ‘whitelisting’ IP addresses for compliance with data protection regulations.

This study was approved by the University of Exeter Sport and Health Sciences Ethics Committee (200 708‐A‐01). All respondents provided consent to participate via a series of check‐boxes, confirming they understood the study and were providing information on behalf of their centre.

Data are presented as frequency statistics, and free‐text responses are provided to emphasise predominant themes within responses.

## 3 RESULTS

The survey was completed by *n* = 31 respondents from across the United Kingdom, representing specialist (*n* = 24; ~50% of specialist U.K. centres) and network (*n* = 7) centres, covering adult (*n* = 11), paediatric (*n* = 16) and mixed (*n* = 4) care centres. In total, *n* = 27 respondents were physiotherapists (lead CF specialist, *n* = 15; CF specialist, *n* = 11; non‐CF specialist, *n* = 1) within their respective CF MDTs. The remaining responses were completed by Therapy Assistants/Technicians (*n* = 2), Exercise Therapist (*n* = 1) and Exercise Practitioner (*n* = 1).

**FIGURE 1:**
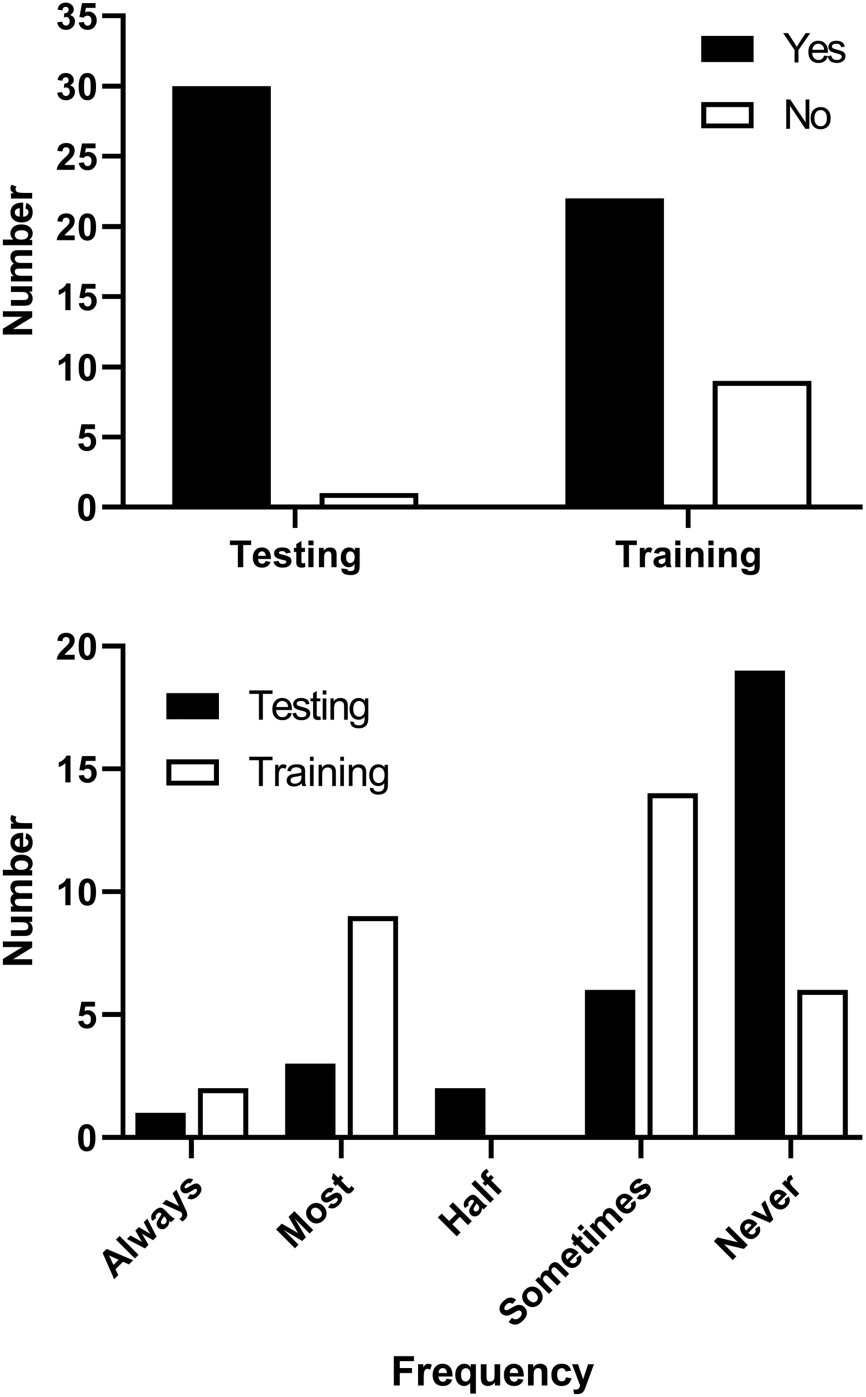
Number of responses to Questions 1 and 4 (*Has the COVID‐19 pandemic affected your ability to deliver exercise testing/training?*) and 2 and 5 (*How often are you able to undertake exercise testing/training due to the pandemic?*)

The majority of respondents stated the pandemic restricted their ability to undertake exercise testing (97%) and exercise training (71%), with the relative frequency of both being negatively affected (Figure 1).

Free‐text responses to questions related to adaptation of practices, barriers and beneficial resources highlighted a number of common themes (Table 2). A large proportion of respondents (*n* = 22, 71%) stated that they would maintain some form of telehealth (e.g., delivery of classes, home monitoring and consultations) within their clinical practice (Table 3), although it is feasible that some may have already been using online exercise classes as part of their practice (Table 2).

**TABLE 2:** Common themes highlighted in free‐text responses to questions related to adaptation, barriers and beneficial resources

Question	Responses
How has you centre adapted exercise testing in light of the pandemic?	Stopped some testing modalities (*n* = 23)^a^
	Inpatient testing only (*n* = 6)
	Virtual/home‐based testing (*n* = 5)
How has your centre adapted exercise testing in the light of the pandemic?	Video/online classes/sessions (*n* = 22)
	Inpatient training (*n* = 5)
	Stopped altogether (*n* = 2)
	Home visits (*n* = 1)
	Outdoor exercise (*n* = 1)
What have been the major barriers to delivering exercise services (testing and training) during the pandemic?	Access to patients (*n* = 12)
	Reduced staffing (*n* = 10)
	Access to facilities/space (*n* = 9)
	Concern around exercise as an aerosol generating procedure (*n* = 5)
	Local and national restrictions (e.g., movement/home visits/distancing) (*n* = 5)
What resources have you found to benefit your team in during the pandemic?	Improved IT facilities and access to online platforms (*n* = 13)
	Existing online classes/resources (*n* = 10)
	Additional finances made available for equipment (*n* = 3)

^a^ Answers are not mutually exclusive and respondents may have stated more than one thematic reason/response. A full list of free‐text responses in provided in File S1.

**TABLE 3:** Selected free‐text responses to Question 9: ‘Are there any changes you have made due to the pandemic that you intend to keep and/or maintain?’, with telehealth highlighted as a predominant thematic response

Telehealth	‘Continue doing exercise classes through video’.
	‘IT and video consultation support’.
	‘Continue to do online live and on demand classes for the foreseeable future’.
	‘More virtual exercise sessions with patients at home via Attend Anywhere’.
	‘Virtual Leisure Centre classes’.
	‘Some video calls for patients who cannot attend in person’.
	‘Virtual exercise support’.
	‘We will keep doing virtual classes’.
	‘Video consultations, remote lung function monitoring’.
	‘More virtual/video reviews as needed’.
	‘Video clinics and spirometry’.
	‘Yes, the video sessions have been a great success’.
	‘Will keep virtual exercise training sessions’.
	‘Video monitoring and calling’.
	‘Some video conferencing. This enables you to see the house and be realistic about what can be done in the home’.
	‘Retain video calls’.
	‘Video calls for all patients’.
	‘Using video calling systems more in the future’.
	‘Keep: ‐ video clinics and exercise reviews ‐ Chester Step Test as an extra exercise testing modality’.
	‘Continue with virtual sessions. Hoping to provide group sessions’.
	‘Would continue with some patients doing exercise sessions via video calls’.
Other responses	‘Continue with most of changes, hope to reintroduce exercise testing. Looking at sit to stand test’.
	‘I would intend to keep the weekly exercise programming going maybe in school holidays or especially while gyms are shut, but redeployment will not allow this to continue, but could be reinstated when we start to return to normal’.
	‘Polar coach. Looking to set up team Strava account if possible in line with trust regulations. We are also looking into the use of social media to help promote exercise and our exercise groups’.
	‘None from an exercise viewpoint’.

*Note*: Responses are provided as written by respondents, with only spelling mistakes changed to increase readability. A number of single word responses (e.g., ‘videos’) have been omitted but counted in statistics. Free‐text responses with identifying information have been removed.

Patients asked their MDTs a variety of questions about exercise and CF, with safety surrounding exercise and ideas for exercises cited as common questions (File S1). The majority of respondents (*n* = 27) felt they were able to confidently answer these questions. Respondents themselves highlighted a number of questions and comments, across numerous themes, including short‐term and long‐term care for pwCF, guidelines for ongoing practice and the changing nature and direction of engagement with exercise (File S1).

## 4 DISCUSSION

This is the first evaluation of how the COVID‐19 pandemic has impacted delivery of exercise services in U.K.‐based CF centres. Our findings demonstrate that, although exercise testing services have been drastically reduced, exercise training provision has adapted and has continued to be offered in novel ways, particularly through increased use of digital health technology.

It is clear from our representative group of CF centres that many have adapted their clinical practice in multiple ways, with routine use of video calling being predominantly noted. The use of telehealth to deliver exercise training for pwCF prior to the pandemic was previously shown to be feasible,^8^ and delivery of exercise services using virtual platforms during the pandemic has since been described anecdotally^9^ and evaluated^10^ within individual U.K. MDTs. The present survey shows promising implementation of this practice for the benefit of pwCF and further notes that many centres plan to continue utilising online resources for clinical practice.

Aside from exercise services, telehealth has emerged as a promising tool for delivery of CF care, being perceived as feasible and acceptable to patients and clinicians alike.^11^, ^12^ Moreover, individual CF centres have detailed the changing practices, acceptance and challenges of integrating telehealth into clinics,^13^, ^14^ and therefore, it should be acknowledged that the adoption of telehealth services within CF will also be associated with numerous financial, ethical and local and national regulatory challenges. For example, equity of access to sufficient internet services and technology (e.g., computers and smartphones), patient access to home monitoring devices (e.g., spirometers), clinician access to patients (i.e., for physical examination and collection of sputum and blood samples) and compliance with digital regulations (e.g., generation, transfer and storage of data) will all pose logistical challenges for patients and clinical teams alike to overcome.^15^, ^16^, ^17^

The adoption of digital services aligns with the long‐term NHS strategy of utilising more technology in routine care,^18^ with the recent ‘Carter Report’; reviewing efficiency and productivity within the NHS; and highlighting the need for enhanced digital solutions.^19^ The present findings that CF MDTs are currently adapting practice and using digital tools, with a view to long‐term adoption of these services, is therefore encouraging.

However, as noted within free‐text responses, CF MDTs face continued challenges concerning finances, staffing, equipment and space in order to ensure services are maintained. Therefore, individual CF MDTs, NHS Trusts and Clinical Commissioning Groups must be aware of, and adequately address, these challenges to ensure successful continuation of adapted services.

## CONFLICT OF INTEREST

There are no conflicts of interest to report.

## AUTHOR CONTRIBUTIONS

All authors conceived and designed the study; OWT and CAW coordinated delivery of survey and collation of results; OWT analysed results and drafted the manuscript; all authors critically revised and approved final manuscript for publication.

## ETHICS STATEMENT

This study was approved by the University of Exeter Sport and Health Sciences Ethics Committee (200 708‐A‐01). All respondents provided consent to participate via a series of check‐boxes, confirming they understood the study and were providing information on behalf of their centre.

## Supporting information


**Data S1.** Supporting information

## Data Availability

The data that support the findings of this study are available on request from the corresponding author. The data are not publicly available due to privacy or ethical restrictions.
